# SPR Distance Computation for *Unrooted* Trees

**DOI:** 10.4137/ebo.s419

**Published:** 2008-02-09

**Authors:** Glenn Hickey, Frank Dehne, Andrew Rau-Chaplin, Christian Blouin

**Affiliations:** 1 School of Computer Science, Carleton University, Ottawa, Canada K1S 5B6; 2 School of Computer Science, Carleton University, Ottawa, Canada; 3 Faculty of Computer Science, Dalhousie University, Halifax, Canada; 4 Faculty of Computer Science, Dalhousie University, Halifax, Canada

**Keywords:** unrooted trees, SPR distance, lateral gene transfer, phylogenetic tree metrics

## Abstract

The subtree prune and regraft distance (*d_SPR_*) between phylogenetic trees is important both as a general means of comparing phylogenetic tree topologies as well as a measure of lateral gene transfer (LGT). Although there has been extensive study on the computation of *d_SPR_* and similar metrics between *rooted* trees, much less is known about SPR distances for *unrooted trees*, which often arise in practice when the root is unresolved. We show that unrooted SPR distance computation is NP-Hard and verify which techniques from related work can and cannot be applied. We then present an efficient heuristic algorithm for this problem and benchmark it on a variety of synthetic datasets. Our algorithm computes the *exact* SPR distance between unrooted tree, and the heuristic element is only with respect to the algorithm’s computation time. Our method is a heuristic version of a fixed parameter tractability (FPT) approach and our experiments indicate that the running time behaves similar to FPT algorithms. For real data sets, our algorithm was able to quickly compute *d_SPR_* for the majority of trees that were part of a study of LGT in 144 prokaryotic genomes. Our analysis of its performance, especially with respect to searching and reduction rules, is applicable to computing many related distance measures.

## 1. Introduction

Phylogenetic trees are used to describe evolutionary relationships. DNA or protein sequences are associated with the leaves of the tree and the internal nodes correspond to speciation or gene duplication events. In order to model ancestor-descendant relationships on the tree, a direction must be associated with its edges by assigning a root. Often, insufficient information exists to determine the root and the tree is left unrooted. Unrooted trees still provide a notion of evolutionary relationship between organisms even if the direction of descent remains unknown.

The phylogenetic tree representation has recently come under scrutiny with critics claiming that it is too simple to properly model microbial evolution, particularly in the presence of lateral gene transfer (LGT) events (Doolittle, 1999). A LGT is the transfer of genetic material between species by means other than inheritance and thus cannot be represented in a tree as it would create a cycle. The prevalence of LGT events in microbial evolution can, however, still be studied using phylogenetic trees. Given a pair of trees describing the same sets of species, each constructed using different sets of genes, a LGT event corresponds to a displacement of a common subtree, referred to as a SPR operation. The SPR distance is the minimum number of SPR operations, denoted by *d_SPR_*, that explain the topological differences between a pair of trees. It is equivalent to the number of transfers in the most parsimonious LGT scenario (Beiko and Hamilton, 2006). In general, *d_SPR_* can be used as a measure of the topological difference between two trees, e.g. for comparing the outputs of different tree construction algorithms.

Tree bisection and reconnection (TBR) is a generalization of SPR that allows the pruned subtree to be rerooted before being regrafted. Computation of the TBR distance (*d_TBR_*) was shown to be NP-hard (nondeterministic polynomial-time hard) by Allen and Steel (2001), who also provided two rules that reduce two input trees to a size that is a linear functions of *d_TBR_* without altering their distance. These rules, which reduce common chains and subtrees, also form the basis of algorithms that compute the SPR distance between rooted trees (*d_rSPR_*) (Bordewich and Semple, 2004) as well as hybridization number (*h*) (Bordewich et al. 2007), see Section 3.3. Such algorithms proceed as follows. First the distance problem is shown to be equivalent to counting components of a maximum agreement forest, and then it is shown that the application of the rules do not alter the number of components in the forest. These steps have been successfully applied to *d_TBR_*, *d_rSPR_* and *h* but not *d_SPR_*, for which no equivalent agreement forest problem is known. As a consequence, the computational complexity of *d_SPR_* has remained an open problem. We provide a proof of NP-Hardness in Section 2. In Section 3, we present an efficient algorithm that relies only on the subtree reduction rule to compute the SPR distance of unrooted trees. An implementation of this algorithm was tested on a variety of data, and the results are analyzed in Section 4. In particular, we show that the conjecture that chain decomposition is *d_SPR_*-preserving for unrooted trees (Allen and Steel, 2001) is strongly supported by our data.

## 2. SPR Distance Computation is NP-Hard for Unrooted Trees

Hein et al. (1996) showed that computing the size of a the Maximum Agreement Forest (MAF) of two trees is NP-Hard by reducing it from Exact Cover of 3-Sets (X3C). Later, Allen and Steel (2001) proved that this result is insufficient to show the hardness of unrooted SPR distance because there is no direct relationship between MAF size and *d_SPR_*, as was previously claimed. Similar techniques have since been used by Bordewich and Semple (2004) to show that rooted SPR distance is NP-Hard via reduction from X3C to a rooted version of MAF. We show that although *d_SPR_* cannot be used to compute *| MAF |* in general, it can for the trees used in the polynomial-time reduction from X3C and this is sufficient to show that *d_SPR_* is NP-Hard as well. We begin with two preliminary definitions.

### Definition 2.1

An *agreement forest* for two trees is any common forest that can be obtained from both trees by cutting the same number of edges from each tree, applying forced contractions after each cut. A *maximum agreement forest* (MAF) for two trees is an agreement forest with a minimum number of components. (Hein et al. 1996)

### Definition 2.2

The *exact cover by 3*-*sets* (X3C) problem is defined as follows (Garey and Johnson, 1979): Given a set *X* with *|X|* =*n* = 3*q* and a collection *C* of *m* 3-element subsets of *X*. Does *C* contain an exact cover for *X,* i.e. a sub-collection *C*′ ⊆ *C* such that every element of *X* occurs in exactly one member of *C*′ ?

*NOTE*: This problem remains NP-Complete if no element occurs in more than three subsets. Also note that this problem remains NP-Complete if each element occurs in *exactly* three subsets. This second property is implied by Hein et al. (1996) though never explicitly stated. A supplemental proof is provided in Appendix A.

**Figure 1:**
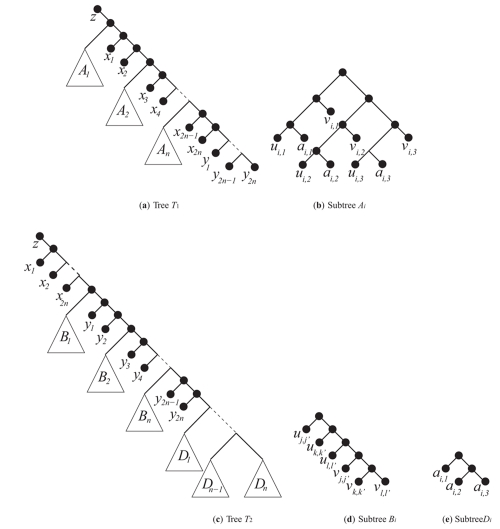
Reduction of an instance of X3C to *|MAF*(*T*1*,T*2) *|* from an {*a,u,v*} triplet. The instance of X3C has a solution if and only if *|MAF* (*T*1*,T*2) *|* = 20*q* + 1 (where *n* = 3*q*).

We now review the polynomial-time reduction from X3C to MAF provided by Hein et al. (1996), clarifying their notation to refl ect that each element of *X* belongs to *exactly* three subsets in *C*, i.e. *|X|* =*|C|* =3*q* = *m* = *n*, a fact implied but not clearly stated in their paper. An instance of X3C is transformed into two rooted phylogenetic trees shown in Figure 1. Each element of *X* is represented by a triplet of the form {*a, u, v*}and each triplet appears 3 times in each tree, once for each occurrence in a subset in *C*. Tree *T*_1_ is illustrated in Figure 1(a). Each subtree *A_i_* ∈ *T*_1_, shown in Figure 1(b) corresponds to a subset *c_i_* ∈ *C*. Each subtree of *A_i_* induced by the triple {*a_i,j_, u_i,j_, v_i,j_*}where *j* ∈ {1, 2, 3} corresponds to a single element of *X*.

Tree *T*_2_, shown in Figure 1(c), has the same leaf set as *T* _1_ but a different topology. Each *D_i_* subtree of *T* _2_, as seen in Figure 1(e), corresponds to a subset in *C* except only the *a*-part of each triplet is present. Each *B_i_* subtree of *T* _2_, as seen in Figure 1(d), corresponds to an element in *X*. Each such element *x* = {*a*, *u*, *v*} in the set *X* appears in three different subsets of *C: c_j,_* *c_k,_* and *c_l._* Without loss of generality, assume it consists of the first element of *c_j_*, second element of *c_k,_* and third element of *c_l_*. The corresponding *B* tree would have leaves {*u_j_*_,_*_j_*_′_*_,_* *u_k_*_,_*_k_*_′_*_,_* *u_l_*_,_*_l_*_′_*_,_* *v_j_*_,_*_j_*_′_*_,_* *v_k_*_,_*_k_*_′_*_,_* *v_l_*_,_*_l_*_′_} where *j*′ = 1, *k*′ = 2, *l*′ = 3.

(Hein et al. 1996) show that *|MAF*(*T*_1_*, T*_2_)*|* = 20*q* + 1 if and only if *C* contains an exact cover of *X*. Note that we have added the *z* leaf to these trees, unrooting them. This does not have any affect on the *|MAF|* as *z* can remain attached to *x*_1_ in the agreement forest without the addition of any new components.

Proving that *d_SPR_*(*T*_1_*, T*_2_) = *|MAF*(*T*_1_*, T*_2_) − 1*|* is sufficient to transform any instance of X3C where |*X|* =*|C|* =3*q* to an instance of *d_SPR_*. In fact, it is sufficient to show that the inequality d*_SPR_*(*T*_1_, *T*_2_) ≤ *|MAF*(*T*_1_*, T*_2_) − 1*|* is true as *d_SPR_*(*T*_1_*, T*_2_) ≥ *|MAF*(*T*_1_*, T*_2_) − 1*|* follows from Lemma 2.7(b) and Theorem 2.13 from (Allen and Steel, 2001). We proceed much in the same way as the original proof, noting that each SPR operation used to transform to *T*_1_ to *T*_2_ corresponds to a cut required to form their MAF.

*MAF*(*T*_1_*, T*_2_) is formed by the cutting edges from *A_i_* subtrees (and the corresponding subtrees in *T*_2_) in either of two possible ways (Hein et al. 1996):

1. Cut leaves *u_i,_*_1_*, v_i,_*_1_*, u_i,_*_2_*, v_i,_*_2_*, u_i,_*_3_*, v_i,_*_3_ and then prune the remaining subtree formed by leaves {*a_i,_*_1_*, a_i,_*_2_*, a_i,_*_3_}. Such a procedure contributes 7 components to the MAF.
2. Cut the leaves *a_i,_*_1_*, a_i,_*_2_*, a_i,_*_3_ then cut each of the remaining two-leaf subtrees: {*u_i_,*_1_*, v_i_,*_1_}, {*u_i,_*_2_*, v_i,_*_2_}, and {*u_i,_*_3_*, v_i,_*_3_}. These operations contribute 6 components to the MAF.

We now show that given two trees *T*_1_ and *T*_2_ and their MAF, which was created using the above cut operations, there exists *|MAF|* − 1 SPR operations that can transform *T*_1_ to *T*_2_. In particular, for each set of cut operations, there exists an equivalent set of SPR operations.

1. Prune leaves *u_i,_*_1_*, v_i,_*_1_*, u_i,_*_2_*, v_i,_*_2_*, u_i,_*_3_*, v_i,_*_3_ from *A_i_* and regraft them onto the chain, forming *B_i_* subtrees in the required positions. Prune the subtree {*a_i,_*_1_*, a_i,_*_2_*, a_i,_*_3_} and regraft into the position of *D_i_*. In this case, 7 SPR operations are performed.
2. Prune the leaves *a_i,_*_1_*, a_i,_*_2_*, a_i,_*_3_ and regraft them onto the chain, forming a *D_i_* subtree in the proper position. Prune the remaining two-leaf subtrees: {*u_i,_*_1_*, v_i,_*_1_}, {*u_i,_*_2_*, v_i,_*_2_}, and {*u_i,3_, v_i,3_*} and regraft them onto the chain, forming *B_i_* subtree components in the required position. 6 SPR operations are used.

There is a one-to-one correspondence between cuts formed when creating the MAF and SPR operations that can transform *T*_1_ to *T*_2_. Thus *d_SPR_*(*T*_1_, *T*_2_) ≤ *|MAF*(*T*_1_, *T*_2_)*|* − 1 and the proof is completed.

## 3. Algorithm for *^d^SPR* Computation

### 3.1. Definitions

**Figure 2:**
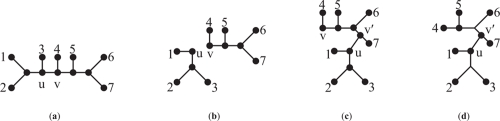
2(**a**) Original tree. 2(**b**) Edge *uv* is removed, pruning subtree rooted at *u*. 2(**c**) Subtree is regrafted, creating new vertex *V*′. 2(**d**) Degree-2 vertex *v* is contracted.

All trees referred to in this paper, unless otherwise stated, are unrooted binary phylogenetic trees. Such trees have interior vertices of degree 3 and uniquely labeled leaves. Given a tree *T*, let *V* (*T* ), *L* (*T* ) and *E* (*T* ) ∈{*V* (*T*) × *V* (*T* )} be the vertex, leaf, and edge sets of *T* respectively. A tree can be rooted by adding a root vertex of degree 2. A pendant subtree of *T* is any rooted tree *T*′ such that *V*(*T*′) ⊆ *V*(*T*)*, L*(*T*′ ) ⊆ *L*(*T* ) and *E*(*T*′ ) ⊆ *E*(*T* ). A SPR operation on a tree *T* is defined by the following three steps, illustrated in Figure 2. First, an edge {*u, v*} ∈ *E*(*T* ) is removed, effectively pruning a pendant subtree rooted at *u* from *T*. A new interior vertex *w* is created by subdividing an edge in *T* and the subtree is then regrafted by creating edge {*u, w*}. Finally, the degree-2 vertex *v* is contracted by identifying its incident edges. The SPR distance between *T*_1_ and *T*_2_, denoted *d_SPR_*(*T*_1_*, T*_2_), is the minimum number of SPR operations required to transform *T*_1_ into *T*_2_. Furthermore, *d_SPR_* is a metric (Allen and Steel, 2001).

### 3.2. Exhaustive search

The reduction rules referred to above only serve to transform the original problem into smaller subproblems. These subproblems must still be solved with an exhaustive search as the problem is NP-Hard (see proof in Appendix). Let *G_SPR_*(*n*) be the graph such that each vertex in the graph is associated with a unique tree topology with *n* leaves, and all possible topologies are in the graph. A pair of vertices in this graph are connected if their SPR distance is 1. Computing *d_SPR_*(*T*_1_*, T*_2_) is therefore equivalent to finding the length of the shortest path between *T*_1_ and *T*_2_ on *G_SPR_*(*n*) and can be computed through an exhaustive breadth-first search beginning at *T*_1_. Allen and Steel (2001) showed that each tree will have *O*(*n*^2^) neighbors in the graph and it follows that the search will visit *O*(*n*^2^) trees of distance 1 from *T*_1_, *O*(*n*^4^) trees of distance 2, up to *O*(*n*^2^*^k^*) trees of distance *k*. A hash table is kept to ensure the same tree is not visited twice. Assuming that it can be updated in constant time, each tree can be processed in *O*(*n*) bringing the time and space complexity of the search to *O*(*n*^2^*^k^*^+1^).

While it is still an open problem to determine if reduction rules can be found to reduce *n* to *k* in the asymptotic complexity above, the value of the exponent can be reduced significantly. Observe that there must be some tree *T*′ such that *d_SPR_*(*T*_1_*, T*′) = ⌊*k/*2⌋ and *d_SPR_*(*T*_2_*, T*′) = ⌈ *k/*2*T*′⌉ because *d_SPR_* is a metric and therefore satisfies the triangle inequality. *T*′ and, correspondingly, *k* can be computed by performing two breadth-first searches, with origins at *T*_1_ and *T*_2_ simultaneously. During the *ith* iteration of the search, all trees of distance *i* from first *T*_1_ then *T*_2_ are explored and updated into the same hash table. *T*′ is the first tree to be found by both searches and *d_SPR_*(*T*_1_*, T*_2_) is 2*i* − 1 if *T*′ is found in the search for *T*_1_ or 2*i* otherwise. Pseudocode is given in Algorithm 1. The time complexity of this algorithm is *O*(*n*^⌊^*^k/^*^2⌋+1^) + *O*(*n*^⌈^*^k/^*^2⌉+1^) = *O*(*n^k^*^+2^). This is a significant reduction from the simple search but the complexity is still prohibitive. Fortunately, heuristics can greatly speed up many instances of the problem while still guaranteeing an exact answer.

**Algorithm 1** SPRDIST (*T***1*,*** *T***2**)

1: **if** *T*_1_ = *T*_2_ **then**

2:  return 0

3: **end if**

4: Apply subtree reductions to *T*_1_ and *T*_2_

5: *d* ← 0

6: *H* ← empty hash table

7: *L*_1_*, L_A_* ← empty lists

8: Insert *T*_1_ into *L*_1_

9: Insert *T*_2_ into *L_A_*

10: **loop**

11:  *L*_2_*, L_B_* ← empty lists

12:  **if** ITERATE(*L*_1_, *L*_2_, *H*, *T*_2_) = TRUE **then**

13:   **return** *d*

14:  **else**

15:   *L*_1_ ← *L*_2_

16:   *d* ← *d* + 1

17:  **end if**

18:  **if** ITERATE(*L_A_*, *L_B_*, *H*, *T*_1_) = TRUE **then**

19:   **return** *d*

20:  **else**

21:   *L_A_* ← *L_B_*

22:   *d* ← *d* + 1

23:  **end if**

24: **end loop**

### 3.3. Heuristic improvements

**Figure 3:**
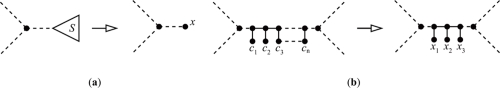
Reduction rules applied to a tree. 3(**a**) A subtree is reduced to a leaf. 3(**b**) A chain of length *n* is reduced to a chain of length 3.

A subtree reduction replaces any pendant subtree that occurs in both input trees by a single leaf with a new label in each tree as as shown in Figure 3(a). A chain reduction, illustrated in 3(b), replaces any chain of pendant subtrees that occur identically in both trees by three new leaves with new labels correctly oriented to preserve the direction. Allen and Steel (2001) showed that maximum application of both of these rules reduces the size of the input trees to a linear function of *d_TBR_*. This result also holds for *d_SPR_* as *d_SPR_* ≤ 2*d_TBR_* for two trees since each TBR operation can be replaced by 2 SPR operations. It is trivial to show that subtree reductions do not alter *d_SPR_* but, unlike *d_TBR_* it is presently unknown whether or not chain reductions affect *d_SPR_*, therefore they can not be used in an exact algorithm. However, our experimental results, further described in Section 4, do support the conjecture that chain reductions do not affect SPR distance.

**Algorithm 2** ITERATE (*L_in_***,** *L_out_***,** *H***,** *T* )

1: **for all** *t* ∈ *L_in_* **do**

2:  **if** t ∈ *H* **then**

3:   **return** TRUE

4:  **else**

5:   Append set of SPR neighbors of *t* to *L_out_*

6:   Insert *t* into *H*

7:  **end if**

8: **end for**

9: **return** FALSE

In addition to applying reductions on the input trees, intermediate trees visited during the breadthfirst search can be likewise reduced. For example, if T* is a tree found on the *ith* iteration from *T*_1_ that has a common pendant subtree with *T*_2_, then that subtree can be reduced to a leaf in T* and *T*_2_ without affecting *d_SPR_*(T*, T_2_). Accordingly, the shortest path from *T*_1_ to *T*_2_ will still be found by a search that applies subtree reductions to the intermediate trees. For ease of maintaining the hash table of trees visited, in our implementation we fl ag common subtrees rather than remove them and use these fl ags to avoid performing SPR operations that would prune from or regraft to fl agged subtrees. This process has no adverse effect on the asymptotic complexity of the search as common subtrees and chains can be detected in *O*(*n*). It is expected that performing reductions on intermediate trees will lessen the total number of trees searched but we are unable to show that it will affect the worst case complexity.

Because the number of trees visited in each iteration of the exhaustive search increases exponentially, the asymptotic complexity is bounded by the number of trees explored in the final iteration. It follows that the order in which these trees are searched can have a critical impact on the running time. We attempt to increase the probability that the tree upon which the search is completed is visited near the beginning of an iteration by sorting the trees in each iteration according to how many leaves are eliminated in by subtree reduction. Our hypothesis is that trees with larger common subtrees are more likely to be near the destination tree. Since at most *n* leaves can be eliminated by subtree reductions, the trees can be bucket sorted in *O*(*n*) time, leaving the asymptotic complexity unchanged. These last two heuristics are employed by replacing the call to ITERATEinSPRDISTtoacalltoSORT ITERATE, shown in Algorithm 3.

**Algorithm 3** SORT_ITERATE (*L_in_*, *L_out_*, *H***,** *T*)

1: **for all** *t* ∈ *L_in_* **do**

2:  Flag all subtrees in *t* that also occur in *T*

3: **end for**

4: Bucket Sort *L_in_* in decreasing order by number of vertices fl agged

5: **for all** *t* ∈ *L_in_* **do**

6:  **if** *t* ∈ *H* **then**

7:   **return** TRUE

8:  **else**

9:   Append set of SPR neighbors which preserve fl agged subtrees of *t* to *L_out_*

10:  Insert *t* into *H*

11: **end if**

12: **end for**

13: **return** FALSE

**Figure 4:**
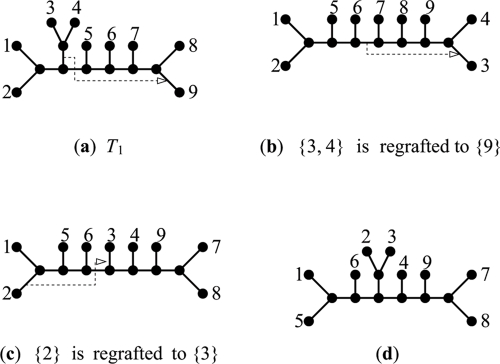
Example of trees whose common clusters cannot be maintained by a minimal SPR path. *T*1 4(a) and *T*2 4(b) have a SPR distance of three but all possible sequences of SPR operations of this length (one is shown by the dotted lines) break the common cluster {7, 8, 9}.

A cluster is the leaf set of a pendant subtree. *T*_1_ and *T*_2_ share a common cluster *C* if they contain pendant subtrees *S*_1_ and *S*_2_ respectively such that *L*(*S*_1_) =*L*(*S*_2_) =*C*. Baroni et al. (2006) showed that the hybridization number of two trees is equal to the total of the hybridization numbers of all their pairs of maximal common clusters. Beiko and Hamilton (2006) made a similar assumption in their heuristic algorithm to measure LGT. Such a decomposition makes intuitive sense for exact SPR distance as well, as it would seem that any SPR operation that affects more than one common cluster would not reduce the distance and therefore not be part of an optimal solution. Unfortunately, this is not the case as evidenced by the counterexample given in Figure 4 which presents *T*_1_ and *T*_2_ that share the common cluster {7, 8, 9}. *d_SPR_*(*T*_1_*,T*_2_) = 3 and 3 SPR operations are shown that transform *T*_1_ into *T*_2_, the first of which breaks the common cluster. Indeed an exhaustive simulation showed that no 3 sequential SPR operations exist to transform the trees that do not break the common clusters. This can be more easily seen by observing that any such sequence would have to regraft 7 to 9 and only 2 operations would be left to transform the cluster {1,2,3,4,5,6} which is clearly insufficient.

## 4. Experimental Results

### 4.1. Datasets

**Figure 5:**
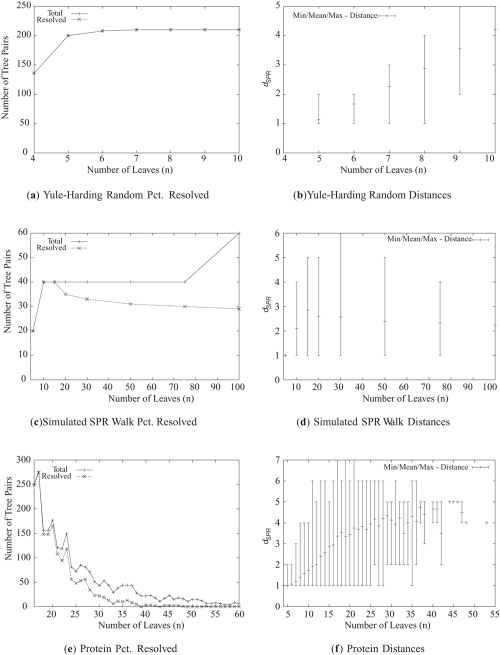
Size, success rate and distance distributions for each dataset. For the protein data, no trees of size greater than 60 were resolved.

The datasets were chosen to analyze the merits of the heuristics discussed in the previous section as well as evaluate our algorithm in a realistic setting. To these ends, we bench-marked our algorithm on a variety of randomly generated trees, as well as trees created by Beiko et al. (2005) in the course of analyzing the proteins from the 144 sequenced prokaryotic genomes available at the time. Two sets of random trees were generated, one by the Yule-Harding model and the other by random walks. Yule-Harding trees are constructed by first creating an edge between two randomly selected leaves, then randomly attaching the remaining leaves to the tree until none are left. The random walk dataset consists of pairs of trees such that one of which is generated by the Yule-Harding model and the other is created from the first by applying a sequence of between 2 and 8 random SPR operations (Beiko and Hamilton, 2006). The size of the datasets, along with the average distances computed by our algorithm are presented in Figure 5. In some cases, the program ran out of memory before finding the solution. The fraction of instances successfully resolved for each type of data is listed in the “% Resolved” column (Fig. 5(a), 5(c) and 5(e)).

### 4.2. Performance

**Figure 6:**
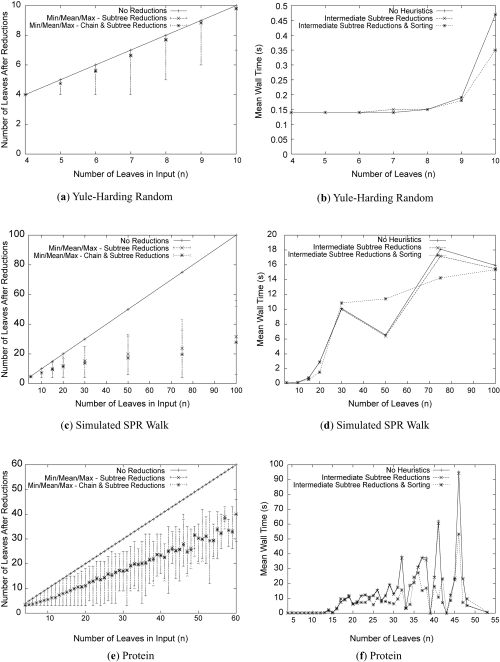
Experimental evaluation of the different heuristics on the three datasets. The effect of the reduction rules on the input tree sizes is displayed on the left. The improvements to the running time made by reducing and sorting intermediate trees are displayed on the right.

The algorithm described in Section 3 was implemented in C++ and benchmarked on a 2.6Ghz Pentium Xeon System with 3G of RAM. The source code is available at http://morticia.cs.dal.ca/lab_public/?Download. This program was executed for all pairs of trees described in Figure 5 with and without the various heuristic optimizations discussed previously. Graphs 6(a), 6(c) and 6(e) in Figure 6 display the effectiveness of the reduction rules’ ability to reduce the input trees. As could be expected, the trees in the protein and random SPR walk datasets are reduced more than the two random datasets as their ratios of size to distance are much higher. In all cases, the amount of reduction increases in correlation to the mean distance rather than *n*. Our method is essentially a fixed parameter tractability (FPT) approach (Downey and Fellows, 1998) and our experiments indicate that the running time behaves similar to FPT algorithms. Also encouraging is the fact that the reduction rules perform much better in practice than the worst-case analysis by Allen and Steel (2001), which predicts a reduction in size to a factor of 28 times the distance. For example, in the random SPR walk dataset whose mean distance is roughly 2, the reductions are effective for *n* > 4 whereas in the worst case it is only guaranteed to work for *n* >= 56. Similar results are visible in the protein dataset graphs as well. As can be seen in these graphs, chain reductions accounts for only a small portion (well under 10%) of the overall gain with subtree reductions making up the rest. We also note that of the roughly 20,000 pairs of trees tested, application of the chain reduction rule did not once affect the SPR distance.

The performance of the remaining heuristics is displayed in terms of running time in graphs 6(b), 6(d) and 6(f) in Figure 6. Applying the reductions to intermediate trees provided very little performance gain, implying that the search space is dominated by trees with few common subtrees and chains. However, sorting the trees visited in each iteration of the search by the number of leaves reduced had a significant impact on the running time for all of the harder cases (*d_SPR_* ≥ 4), speeding up the computation by nearly a factor of 6 for some of the larger protein tree pairs.

## 5. Conclusion

The computation of SPR distances between unrooted phylogenetic trees can be used to compare the evolutionary histories of different genes and provide a lower bound on the number of lateral transfers. Little previous work has been done on this problem though many related tree metrics have been relatively well studied in the literature. The reason for this appears to be primarily due to less insight into the problem’s structure (no known MAF reduction) rather than lack of interest. In this paper we revisited the problem of unrooted SPR distance, showing that it is NP-Hard and providing an optimized algorithm and implementation to solve it exactly. The algorithm is based on dividing the problem into two searches and making use of heuristics such as subtree reductions and reordering. This algorithm was able to quickly compute the exact distance between the majority of proteins belonging to 144 available sequenced prokaryotic genomes and their supertree. Our method can also be used to improve the brute force search component of TBR and rooted SPR distance computation.

Though a polynomial time solution is unlikely due to its NP-Hardness, some possible avenues of future work on this problem remain. One is to show that chain reductions do not affect the distance, a conjecture that is supported by our experimental results but for which an analytical proof remains absent. This result would be sufficient to show that unrooted SPR distance is fixed parameter tractable, being exponential only in terms of the distance and not the size of the trees. Bordewich et al. (2007) used a decomposition by common clusters was used with significant practical success. We showed that such a technique cannot be directly applied to the problem of unrooted SPR distances but perhaps a variation of this technique can.

The contributions of this paper can thus be summarized as follows: (1) We show that SPR distance computation is NP-hard for unrooted trees. (2) We present an efficient heuristic algorithm for this problem and benchmark it on a variety of synthetic datasets. Our algorithm computes the *exact* SPR distance between unrooted trees, and the heuristic element is only with respect to the algorithm’s computation time. Our method is a heuristic version of a fixed parameter tractability (FPT) approach (Downey and Fellows, 1998) and our experiments indicate that the running time behaves similar to FPT algorithms. For real data sets, our algorithm was able to quickly compute *d_SPR_* for the majority of trees that were part of a study of LGT in 144 prokaryotic genomes. (3) Our analysis of its performance, especially with respect to searching and reduction rules, is applicable to computing many related distance measures. (4) In Bordewich et al. (2007), a decomposition by common clusters was used with significant practical success. We show that such a decomposing by common clusters cannot be used to compute exact SPR distance for *unrooted* trees (Fig. 4) which is somewhat counterintuitve.

## Appendix A

### X3C remains NP-complete when each element occurs in exactly 3 subsets

In this appendix we verify that X3C remains NP-Complete in the special case where each element occurs in *exactly* three subsets. Consider an instance of X3C in which no element occurs in more than three subsets. We provide a polynomial time reduction from such an instance, known to be NP-Complete, into an instance in which each element occurs in exactly three subsets. Let:

*Y*_1_ ⊆ *X* : Elements of X that appear in exactly one subset

*Y*_2_ ⊆ *X* : Elements of X that appear in exactly two subsets

*Y*_3_ ⊆ *X* : Elements of X that appear in exactly three subsets

So |*Y*_1_| + 2|*Y*_2_| + 3|*Y*_3_| =|*X*| =3*q*

For each element to appear in exactly three subsets, we must add 2*|Y*_1_*|* + *|Y*_2_*|* elements to subsets in *C*.

Let multiset *Z* = {*z*_0_, *z*_1_, …, z_3_*_p_*_−1_} = *Y*_1_ + *Y*_1_ + *Y*_2_ be these elements we have to add. Note that |*Z*| =3*p* where *p* = 2(*q* − |*Y*_3_|) − |*Y*_2_|.

Let *X*′ = {*x*′_0_, *x*′_1_, …, *x*′_3_*_p_*_−1_} be a set of new elements such that |*X*′*|* = 3*p* and *X* ∩ *X*′ = φ.

We now create a collection *C*′ of new subsets out of *Z* and *X*′ so that each element in *X* ∪*X*′ appears in a subset in *C* + *C*′ exactly three times.

For each *i* = 0, 3*,* …*,* 3*p* − 1, we add four subsets to *C*′:

c′_4_*_i_* ={*x*′*_i_*_,_ *x*′*_i_*_+1_, *x*′*_x_*_+2_}

c′_4_*_i+_*_1_ ={*z**_i_*, *x*′*_i_*, *x*′*_i_*_+1_}

c′_4_*_i+_*_2_ = {*z**_i_*_+1_, *x*′*_i_*_+1_, *x*′*_i_*_+2_}

c′_4_*_i+_*_3_ ={*z**_i_*_+2_, *x*′*_i_*_+2_, *x*′*_i_*}

We now show that *X X*′ and *C* + *C*′ form an instance of X3C such that every element of *X X*′ appears in 3 subsets in *C* + *C*′ and *X* has a cover in *C* if and only if *X X*′ has a cover in *C* + *C*′.

(*if*): If *X* has a cover in *C*, then *X* ∪ *X*′ has a cover in *C* + *C*′: Let *S* ⊆ *C* be the cover of *X*. Then *S* + *c*′_0_+ *c*′_4_+ *c*′_8_+ … + *c*′_12_*_p_*_−1_ is a cover *X* ∪ *X*′.

(*only if* ): If *X* ∪ *X*′ has a cover in *C* + *C*′, then *X* has a cover in *C*: Similar to above, the only way to cover *X*′ is with *c*′_0_+ *c*′_4_+ *c*′_8_+ … + *c*′_12_*_p_*_−1_ and no other elements of *C*′ can be part of an exact cover. This means that *X* is covered entirely by subsets in *C* so *X* is exactly covered by *C*.
